# LncLSTA: a versatile predictor unveiling subcellular localization of lncRNAs through long-short term attention

**DOI:** 10.1093/bioadv/vbae173

**Published:** 2024-11-22

**Authors:** Kai Wang, Yueming Hu, Sida Li, Ming Chen, Zhong Li

**Affiliations:** School of Information Engineering, Huzhou University, Huzhou, Zhejiang 313000, China; School of Science, Zhejiang Sci-Tech University, Hangzhou, Zhejiang 310018, China; College of Life Sciences, Zhejiang University, Hangzhou, Zhejiang 310003, China; College of Life Sciences, Zhejiang University, Hangzhou, Zhejiang 310003, China; College of Life Sciences, Zhejiang University, Hangzhou, Zhejiang 310003, China; School of Information Engineering, Huzhou University, Huzhou, Zhejiang 313000, China; School of Science, Zhejiang Sci-Tech University, Hangzhou, Zhejiang 310018, China; College of Life Sciences, Zhejiang University, Hangzhou, Zhejiang 310003, China

## Abstract

**Motivation:**

Much evidence suggests that the subcellular localization of long-stranded noncoding RNAs (LncRNAs) provides key insights for the study of their biological function.

**Results:**

This study proposes a novel deep learning framework, LncLSTA, designed for predicting the subcellular localization of LncRNAs. It firstly exploits LncRNA sequence, electron-ion interaction pseudopotentials, and nucleotide chemical property as feature inputs. Departing from conventional *k*-mer approaches, this model uses a set of 1D convolutional and maxpooling operations for dynamical feature aggregation. Furthermore, LncLSTA integrates a long-short term attention module with a bidirectional long and short term memory network to comprehensively extract sequence information. In addition, it incorporates a TextCNN module to enhance accuracy and robustness in subcellular localization tasks. Experimental results demonstrate the efficacy of LncLSTA, showcasing its superior performance compared to other state-of-the-art methods. Notably, LncLSTA exhibits the transfer learning capability, extending its utility to predict the subcellular localization prediction of mRNAs, while maintaining consistently satisfactory prediction results. This research contributes valuable insights into understanding the biological functions of LncRNAs through subcellular localization, emphasizing the potential of deep learning approaches in advancing RNA-related studies.

**Availability and implementation:**

The source code is publicly available at https://bis.zju.edu.cn/LncLSTA.

## 1 Introduction

Long-stranded noncoding RNAs (lncRNAs), a class of noncoding RNA molecules with >200 nucleotides, are transcribed from DNA without being translated into proteins (Nojima and Proudfoot 2022). They play pivotal roles in diverse biological processes such as gene regulation, alternative splicing and genomic imprinting (Chen 2022). Emerging evidence from recent studies highlights the crucial involvement of lncRNAs in cellular differentiation, metabolism, and disease pathogenesis (Gil and Ulitsky 2020). Despite being expressed at lower levels compared to mRNAs, lncRNAs exhibit robust tissue-specific expression patterns, underscoring their essential contributions to cell type-specific processes (Sun *et al.* 2018). Considering the molecular functional complexity of lncRNAs, elucidating their subcellular localization becomes imperative for a comprehensive understanding of their biological roles (Moran *et al.* 2012).

LncRNA subcellular localization is closely linked to their functions by facilitating interactions with spatially restricted binding partners. Compared to nuclear lncRNAs involved in transcriptional and chromatin regulation, cytoplasmic lncRNAs more directly control signal transduction, translation, and RNA stability. Localization to distinct organelles like mitochondria and membraneless bodies enables lncRNA participation in related pathways. Splice variants of the same lncRNA can also display distinct localization and oppose each other functionally. In summary, the subcellular niche provides a structural foundation for lncRNAs to interact with specific biological processes and signaling cascades. Because subcellular localization is intricately tied to lncRNA functionality, there is an urgent need to develop tools to map lncRNA subcellular distribution (Bridges *et al.* 2021).

Early methods for subcellular localization of LncRNA are implemented by experimental methods. However, experiment-based methods are easily influenced by experimental conditions and are time-consuming. LncRNA subcellular localization prediction has received extensive attention through the development of various computational models in recent years. For example, Cao *et al.* (2018) proposed a model (lncLocator) using stacked autoencoders to extract 4-mer features and high-level features, feeding them to support vector machine (SVM) and random forest (RF) respectively, and then combining their outputs to obtain the final prediction. Su *et al.* (2018) developed iLoclncRNA, applying 8-mer features to encode lncRNA sequences. Considering that the dimensionality of 8-mer features is too large, it uses a binomial distribution-based feature extraction method to select the optimal features, which are then fed into SVM to obtain final predictions. Gudenas and Wang (2018) proposed DeepLncRNA using 2-, 3-, 4-, and 5-mer features to encode lncRNA sequences and adding additional features (RNA binding motifs and genomic loci). These combined features are then fed into a neural network to obtain the final prediction. Wang *et al.* (2021a,b) developed an integrated SVM model that uses multiple sequence features including *k*-mer, reverse complementary *k*-mer, nucleic acid composition, dinucleotide composition, trinucleotide composition, and k-spacer nucleic acid pairs to predict lncRNA subcellular localization. Zeng *et al.* (2022) designed a DeepLncLoc model, which firstly divides a sequence into a number of consecutive subsequences, then extracts the patterns of each subsequence, and finally combines these patterns to obtain a complete representation of the lncRNA sequence, after which a textual convolutional neural network is used to learn high-level features and perform prediction tasks. Wang *et al.* (2022) developed IDDLncLoc which is based on an unbalanced data distribution framework for LncRNA subcellular localization. It introduces dinucleotide-based auto-crossing covariance features, *k*-mer nucleotide composition features, and composition, transition, and distribution features to encode raw RNA sequences as vectors. Then, binomial distribution feature selection and recursive feature elimination are used to filter out reliable features. In addition, it is customized with strategies of oversampling in mini-batch, random sampling, and stacking ensemble strategies to overcome the data imbalance on the benchmark dataset. Li *et al.* (2024) proposed an SGCL-LncLoc model by using the supervised graph contrastive learning to significantly enhance the prediction accuracy of lncRNA subcellular localization, highlighting the critical role of graph structures in model enhancement. Zeng *et al.* (2023) developed an LncLocFormer model which utilizes the Transformer architecture and introduces a location-specific attention mechanism, effectively addressing the multi-label localization problem and demonstrating its advantage in handling complex data. Li *et al.* (2023) provided a GraphLncLoc model by transforming lncRNA sequences into de Bruijn graphs and applying graph convolutional networks to convert the sequence classification into the graph classification, thereby improving its prediction accuracy and robustness. These models offer novel methods and perspectives for lncRNA subcellular localization prediction. But these approaches demonstrate a lack of focus on the intricate local nucleotide details, such as interactions between adjacent nucleotides, which are crucial for comprehending the functional and structural aspects of biological sequences. Recently, electron-ion interaction pseudopotentials (EIIP) (Wang *et al.* 2020) and nucleotide chemical properties (NCPs) (Yang *et al.* 2023) have achieved significant success in predicting DNA methylation sites and RNA methylation modification sites, respectively. The EIIP focuses on capturing the physicochemical properties of nucleotide sequences by calculating the potential interaction between electrons and ions, reflecting the electronic and spatial characteristics of sequences. NCP, on the other hand, focuses on the chemical attributes of individual nucleotides, which may influence the local structure of nucleotides and their interactions with other molecules. These features effectively capture certain common biological attributes by converting complex biological data into manageable numerical information. Therefore, they demonstrate the versatility in a range of biological tasks, from the methylation site prediction to the subcellular localization prediction. For example, Yang *et al* (2023) proposed a 4mCBERT model which incorporates features including EIIP and NCP. It outperforms other models in predicting 4mC sites and demonstrates the effectiveness and transferability of these features across different species, confirming their universality in various deep learning applications. These feature sets can actually be referred to address the deficiency in biological information in the subcellular localization prediction of lncRNAs.

This article proposes a novel deep learning-based lncRNA subcellular localization predictor (LncLSTA). It firstly uses one-hot encoding combined with EIIP and NCP of each nucleotide as input features. A series of 1D convolution and maxpooling operations replace the commonly used *k*-mer approach to dynamically aggregate features from surrounding nucleotides, without requiring the trimming or padding of lncRNA sequences. Then, it uses a long-short term attention (LSTA) module and bidirectional long and short term memory (Bi-LSTM) network to process sequence information to efficiently extract features about subcellular localization from nucleotide sequences. Finally, it applies the TextCNN module to continuously mine the sequence feature and outputs the final prediction. Various experimental results show that the combination of these features and neural network modules is complementary and can significantly improve its subcellular localization prediction performance of lncRNAs, and even for mRNAs.

## 2 Datasets, materials, and methods

### 2.1 Dataset

We retrieve known lncRNA subcellular localization information from the RNAlocate database (Cui *et al.* 2022), in which 42 190 RNA-related subcellular localization entries are collected. We then generate a benchmark dataset to train and test our model using the following procedure.

1. 2383 lncRNA subcellular localization entries are selected from all RNA-related subcellular localization entries in RNAlocate.
2. For some lncRNAs with multiple entries, we merge them with the same gene name. In addition, we remove lncRNAs that had no sequence information in NCBI (https://www.ncbi.nlm.nih.gov/) and Ensembl (https://www.ensembl.org/index.html).
3. Because most lncRNAs have only one subcellular localization, we select lncRNAs located at only one position for the model construction.
4. The filtered dataset covers seven different subcellular localizations. Two of seven subcellular localizations have only a very small number of samples (<10). We remove the lncRNAs with too few subcellular localization samples.
5. We use CD-HIT (Fu *et al.* 2012) to remove sequences with sequence similarity of 80% or more.

Finally, we construct a benchmark dataset with 842 lncRNAs covering 5 subcellular localizations including Nucleus, Exosome, Cytoplasm, Cytosol, and Ribosome. The data distribution of the processed dataset is shown in Supplementary Fig. S1. It can be seen that the lengths of LncRNAs distributed in Cytoplasm, Cytosol and Exosome are mostly focused between 200 nt and 3000 nt, while the lengths of lncRNAs localized in Nucleus and Ribosome are concentrated between 200 nt and 3000 nt and >12 000 nt.

To evaluate the performance of the prediction model, we use the above nonredundant dataset to construct 5-fold cross-validation datasets by dividing the dataset into 5 folds, each with a similar distribution of subcellular localization categories. These datasets are used for comparison between our model and other methods. All our datasets, including all benchmark datasets of lncRNAs and independent test datasets, are available on our website (https://bis.zju.edu.cn/LncLSTA).

### 2.2 Feature coding

We firstly use the one-hot encoding of nucleotides as one of our input features, namely, nucleotides A, T, C, and G are encoded by four-bit binary vectors as [1,0,0,0], [0,1,0,0], [0,0,1,0], and [0,0,0,1], respectively. In addition, we introduce EIIP (Wang *et al.* 2020) and NCP features (Yang *et al.* 2023) to improve the subcellular localization prediction of LncRNAs. The EIIP feature is originally represented for amino acids in protein sequences and nucleotides in DNA sequences. We find that it is also suitable for the EIIP in RNA nucleotide sequences. Here, we refer to R (Wang *et al.* 2020) and encode nucleotides A, T, C, and G by 0.1260, 0.1335, 0.1340, and 0.0806, respectively.

For the NCP feature, it has successfully been used for the RNA modification site prediction and other related tasks. We also find that this feature is helpful for the subcellular localization prediction of LncRNAs. NCP feature can be represented based on chemical properties of nucleotides such as ring structure (R), functional groups (F), and hydrogen bonds (H) (Yang *et al.* 2023). Here, A, C, G, and T are coded as (1,1,1), (0,1,0), (1,0,0), and (0,0,1), respectively.

Combining above three features, the following expression is used to represent the form of each lncRNA. Therefore, the input sequences are encoded into features with a shape of *L *×* *8, where *L* is the length of lncrRNA sequence.

### 2.3 Deep learning network architecture

**Figure 1.**
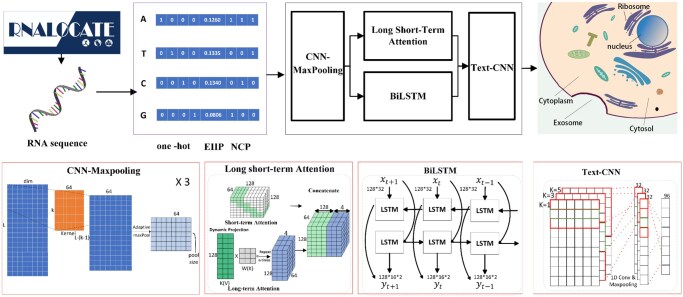
Main LncLSTA framework. In this model, the input is an arbitrary length lncRNA sequence, which is converted into a feature vector of *L *×* *8. This feature vector is fed to the CM module for feature extraction and dimensionality adjustment. After that, the feature vector is input to the sequence information extraction module (LSTA and BiLSTM) and TextCNN to further extract higher-level features. Finally, the LncLSTA model outputs the probability of each category by softmax for classification. The let-top and right-top subfigures are referred from RNAlocate (Cui *et al.* 2022).

The main framework of our LncLSTA is shown in Fig. 1. One advantage of our model is that it can receive different lengths of lncRNA sequences, which does not need to truncate or complement the lncRNA length for the subsequent feature extraction. We first construct a CNN-Maxpooling (CM) module to extract feature information from different combinations of nucleotides. Specifically, we utilize two convolutional kernels (size = 3 and 5, respectively) in parallel and perform three layers of CM operations to obtain two sets of output features in this module. Next, we input both features into the LSTA module and Bi-LSTM network, respectively. Both modules can effectively extract the information contained in the long sequences. Subsequently, feature concatenation is performed based on different kernel settings, and then fed into the Text CNN module, where the high-weight parts of sequences are extracted through the maximum pooling operation. The resulting feature from both pathways passing through the TextCNN module is concatenated and subsequently input into a fully connected network with five neurons, corresponding to five distinct categories of subcellular localization.

#### 2.3.1 CM module

For most of the previous methods, sequence lengths require preprocessing operations such as zero-padding or truncation to ensure each sequence has the same length. However, such preprocessing may result in some sequence lengths being too long due to zero-padding or may cause important information to be lost during truncation. This negatively affects the feature extraction process of the neural network, thereby reducing the accuracy of prediction. Here, we adopt some strategies such as convolution and maxpooling operations to handle the sequence length problem for better utilizing the information in sequences, which can process sequence data with more flexibility.

Before the feature extraction of LncRNA sequence, we adjust the dimensionality order of the input sequence (namely, we swap the feature dimensionality *L *×* *8 and change the feature shape as 8 × *L*), which can facilitate the effective extraction of sequence information using 1D convolution. During the convolution operation, the convolution kernel can slide on the last dimension to achieve the extraction of different combinations of nucleotide information. Subsequently, we perform three consecutive CM operations to extract different combinations of nucleotide information more effectively.

Specifically, we initially set the number of convolution kernels in the first operation to 64, and the feature dimension of each sequence becomes 64 × (*L—*kernel + 1) after the first convolution operation. Then, we perform the maximum pooling operation, and the feature dimension is transformed to 64 × 512. In the second operation, we set the number of convolution kernels to 64 and the dimension of the last dimension after maximum pooling to 256, so the feature dimension is transformed to 64 × 256. During the third operation, we configure the number of convolution kernels to 32, and the maximum pooling dimension to 128. Consequently, the output of final feature dimension after the third operation becomes 32 × 128. Then, we swap the dimension order for the subsequent training, resulting in a feature dimension of 128 × 32. We utilize a straightforward formula to represent the computational model process as

$$y={\mathrm{Conv}}_{3}(\mathrm{Pool}{{(\mathrm{ReLU}(\mathrm{Conv}}_{2}(\mathrm{Pool}(\mathrm{ReLU}(\mathrm{Conv}}_{1}(x)))))))$$

where ${\mathrm{Conv}}_{i}$ indicates the *i*th convolution operation, Pool stands for the pooling operation, and ReLU represents the activation function.

In the subsequent experimental analysis section, we find that such an operation can effectively avoid the use of zero-padding and truncation, thus reducing the loss of information and improving the feature extraction ability of the neural network, which can enhance the prediction accuracy.

#### 2.3.2 Sequence information extraction module

In the feature extraction process, we combine the main idea of long-short (LS) transformer (Zhu *et al.* 2021) with Bi-LSTM network (Huang *et al.* 2015) for learning and training. LS transformer uses a novel long and short term attention mechanism to model long-range correlations by dynamic projection, captures the fine-grained local correlations, and uses a dual normalization strategy to address the scale mismatch between two attention mechanisms. Here, the LSTA mechanism is selected and embedded into our model, and we focus on optimizing the parameters of LSTA to adapt to the dataset and avoid overfitting caused by excessive parameterization. Bi-LSTM is a combination of forward and backward LSTMs and can better capture bidirectional sequence information and semantic dependencies, which is especially suitable for the feature extraction from sequential data such as RNA sequences (Hochreiter and Schmidhuber 1997).

We input the features extracted by the CM module into the LSTA and Bi-LSTM modules. In order to avoid overfitting, we choose 6 attention heads, setting the dropout rate of attention as 0.65 in the learning. Meanwhile, we set the window size of short-term attention to 128 and the projection dimension *r* of long-term attention to 4 to project the 128D feature vector into a lower dimension, thus significantly reduce the spatial complexity. At the same time, we set the hidden layer size of Bi-LSTM to 16 and the dropout to 0.5. Next, we perform a concatenation operation between the matrix outputs from LSTA and Bi-LSTM by splicing the LSTA features 128 × 32 and the Bi-LSTM features 128×(16 × 2) into 128 × 64, thus achieving the integration *y* of features. The detailed process is described as

$$y=h_{t}^{bi}+H_{i,t}$$

 

$$H_{i,t}=\mathrm{softmax}\left[\frac{{Q_{t}W_{i}^{Q}\left[{\tilde{K}}_{t}W_{i}^{K};{\bar{K}}_{i}\right]}^{T}}{\sqrt{d_{k}}}\right]\left[{\overset{ˇ}{V}}_{t}W_{i}^{V};{\tilde{V}}_{i}\right]$$

 

$$h_{t}^{bi}=\left[h_{t}^{f};h_{t}^{b}\right],\;h_{t}=o_{t}\times \tanh\left(C_{t}\right)$$

 

$$C_{t}=f_{t}\times C_{t-1}+i_{t}\times \tanh\left(W_{cx}x_{t}+W_{ch}h_{t-1}+b_{c}\right)$$

where $f_{t}$, $i_{t},\;$and $o_{t}\;$represent the activation of the forget, input and output gate at the time step t, $x_{t}$ denotes the input vector at the time step t, $h_{t-1}$ is the hidden state at the previous time step t−1, $W_{cx}$, and $W_{ch}$ stand for the weight parameters associated with the cell state’s updating operation. $b_{c}$ represents the bias parameter associated with the cell state’s updating operation, $C_{t}$ indicates the cell state at the time step t, $h_{i}^{bi}\;$is the hidden state of the Bi-LSTM at the time step t. $\left[.;.\right]$ denotes concatenating the matrices along the first dimension. Q, K, $V\in R^{n\times d}$ are the query, key and value embeddings, $W_{i}^{Q}$, $W_{i}^{K}$, $W_{i}^{V}\in R^{d\times d_{k}}$ are learned projection matrices.

#### 2.3.3 TextCNN module

After the feature extraction of sequences, we use the TextCNN structure (Guo *et al.* 2019) to predict the subcellular localization of lncRNAs. It extracts the features of sequences through a 1D convolutional layer and a maximum pooling layer. Specifically, we represent the lncRNA sequences as an *L *×* D* matrix, where *D* denotes the feature dimension of each nucleotide. We use three convolutional kernels of different sizes (size = 1, 3, 5, respectively) to aggregate long-range nucleotide features again. This operation integrates the convolution results of three convolution kernels at different positions together, to obtain a more global feature representation. Next, the most significant features on the whole sequence are obtained by the max-pooling operation. Because there are two sets of output features by originally using convolutional kernels of size 3 and 5, two output vectors of the max-pooling layer are sliced together as the input of a fully connected layer with a softmax function to perform the final prediction. The introduction of TextCNN can continuously extract the feature of lncRNA sequences, thereby improving the performance of the prediction accuracy. The detailed process is described as

$$L=\;\mathrm{concatenate}(L_{\mathrm{ou}t^{1}}+L_{\mathrm{ou}t^{2}}+L_{\mathrm{ou}t^{3}})$$

 

$$L_{\mathrm{ou}t^{i}}=\left[\frac{L_{in^{i}}+2\times \mathrm{padding}-\left(\mathrm{kernelsiz}e^{i}-1\right)-1}{\mathrm{stride}}+1\right]\;i=1,2,3$$

where *L* represents the output sequence of TextCNN,$\;L_{\mathrm{out}}$ and $L_{\mathrm{in}}$ denote the output and input size, padding is used to control the size of the output feature map by adding extra pixels or elements around the input data, kernelsize denotes the sliding window size of the convolutional kernel, and stride is the interval at which the convolutional kernel slides across the input data.

### 2.4 Evaluation metrics

We use accuracy (ACC), macro F1-score (maF1), Matthews correlation coefficient (MCC), micro-averaged area under the ROC curve (miAUC), macro-averaged area under the ROC curve (maAUC), and micro-averaged area under the precision–recall curve (miAUPRC) as comprehensive evaluation metrics to assess the performance of LncLSTA in comparison with other methods throughout the experiment.

$$\mathrm{ACC}=\frac{\mathrm{Num}(\mathrm{Pred}=\mathrm{Label})}{\mathrm{Num}(\mathrm{Samples})}$$

 

$$\mathrm{Macro}\;\mathrm{Precisio}n^{\left(i\right)}=\frac{1}{m}\sum_{1}^{m}\frac{TP^{\left(i\right)}}{TP^{\left(i\right)}+FP^{\left(i\right)}}$$

 

$$\mathrm{Macro}\;\mathrm{Recal}l^{\left(i\right)}=\frac{1}{m}\sum_{i=1}^{m}\frac{TP^{\left(i\right)}}{TP^{\left(i\right)}+FN^{\left(i\right)}}$$

 

$$\mathrm{maF}1=\frac{1}{m}\sum_{i=1}^{m}\frac{2\times \mathrm{precisio}n^{\left(i\right)}\times \mathrm{Recal}l^{\left(i\right)}}{\mathrm{precisio}n^{\left(i\right)}+\mathrm{Recal}l^{\left(i\right)}}$$

 

$$\mathrm{MCC}=\frac{TP^{\left(i\right)}\times TN^{\left(i\right)}-FP^{\left(i\right)}\times FN^{\left(i\right)}}{\sqrt{\left(TP^{\left(i\right)}+FP^{\left(i\right)}\right)\left(TP^{\left(i\right)}+FN^{\left(i\right)}\right)\left(TN^{\left(i\right)}+FP^{\left(i\right)}\right)\left(TN^{\left(i\right)}+FN^{\left(i\right)}\right)}}$$

where $TP^{\left(i\right)}$, $FP^{\left(i\right)}$, and $FN^{\left(i\right)}$ denote the ith class’s true positives, false positives, false negatives respectively, and m denotes the number of LncRNA subcellular localization for the classification. MiAUC is calculated by the area under the overall ROC curve using all positive and negative samples across all classes. MaAUC is represented by the average area under the ROC curve for each individual class. MiAUPRC, on the other hand, is computed by the area under the precision–recall curve based on aggregated positive and negative samples across all classes.

### 2.5 Implementation details

In the experiment, the predictor LncLSTA is implemented by Pytorch, and during the model training, the focal loss (Lin *et al.* 2017) is chosen as the loss function. It was originally proposed to solve the problem caused by the imbalance of data. Here, we use the multi-classification focal loss to deal with the lncRNA data for the prediction.

$$\mathrm{Los}s_{\mathrm{class}}=-\frac{1}{m}\alpha y{\left(1-y_{\mathrm{pred}}\right)}^{\gamma }l\mathrm{og}{(y}_{\mathrm{pred}})$$

where m is the number of training samples, y represents the label values, $y_{\mathrm{pred}}$ denotes the predicted label values, α is the scaling factor for class imbalance set to 1 by default, and γ is the focusing parameter set to 2 as referred to the reference (Lin *et al.* 2023). It smoothly adjusts the rate at which easy examples are down-weighted.

In order to extend our model for the prediction of subcellular localization of mRNAs, we generalize the focal loss to the multi-label multi-classification.

$$\mathrm{Los}s_{\mathrm{label}}=\frac{\sum_{i=1}^{n}FL_{i}}{n}$$

where n is the number of species of mRNA subcellular localization. $FL_{i}$ is the ith loss function of subcellular localization, which is defined as

$$FL_{i}=-\alpha_{i}{\left(1-p_{i}\right)}^{\gamma }l\mathrm{og}\left(p_{i}\right)$$

We adjust $\alpha_{i}$ according to each classification of the mRNA dataset, which are set to 0.4, 0.03, 0.92, 0.6, 0.9 and 0.95, respectively. The larger the number of categories, the smaller the corresponding value of parameter${\;\alpha }_{i}$. For γ, we also set it to 2. For each subcellular localization, if the label is 1, then $p_{i}$ is the predicted probability ${\hat{p}}_{i}$. Otherwise, it is $1-{\hat{p}}_{i}$.

$$p_{i}=\left\{\begin{array}{c} {\hat{p}}_{i},\;l\mathrm{abel}=1\\ 1-{\hat{p}}_{i},\;o\mathrm{therwise} \end{array}\right.$$

The value of $p_{i}$ reflects the degree of proximity to the true label or category *y*. The larger the value of $p_{i}$, the closer it is to the category *y*, indicating higher accuracy in classification.

In addition, to avoid overfitting in the deep learning model, we use a dropout of 0.5 for the convolution operation and the fully connected operation, while for the LSTA module, we set a dropout of 0.65, and for the Bi-LSTM network, we set a dropout of 0.5. Before the last fully connected layer, we set a dropout of 0.05. In addition, we use the AdamW optimizer to train the LncLSTA model, with an initial learning rate of 0.0002 and a weight decay of 0.001.

### 2.6 Hyperparameter optimization for LncLSTA

It is important to tune the hyperparameters, which would influence the performance of the prediction model. In our model, we use the grid search algorithm to search for the optimal combination of hyperparameters. Specifically, we focus on hyperparameters such as the size of the convolutional kernel *K*, the number of convolutional kernels *S*, the size of the pooling layer *P*, and the number of attention heads *N*. We choose the size of batch in the range of [128, 256, 512], the size of convolutional kernels in the range of [3,4,5, 6,7], the number of convolutional kernels in the range of [32,64,128], the pooling layer size in the range of [64,128,256], and the number of attention heads in the range of [2,4,6,8].

Through the experiment, we finally determine the best performing hyperparameter combination when the batch is 256, the convolutional kernel size is a combination of 3 + 5. The number of convolutional kernels in the CM module is chosen as 32, 64, and 64 (a detailed selection analysis is described in Supplementary Fig. S2), the dimension for adaptive maxpooling after the first convolution, the second convolution and the third convolution are set to 512, 256, and 128, respectively. The detailed selection discussion about the number of attention heads in the LS term attention module is shown in Supplementary Fig. S3.

## 3 Experimental results

### 3.1 Ablation experiments

**Figure 2.**
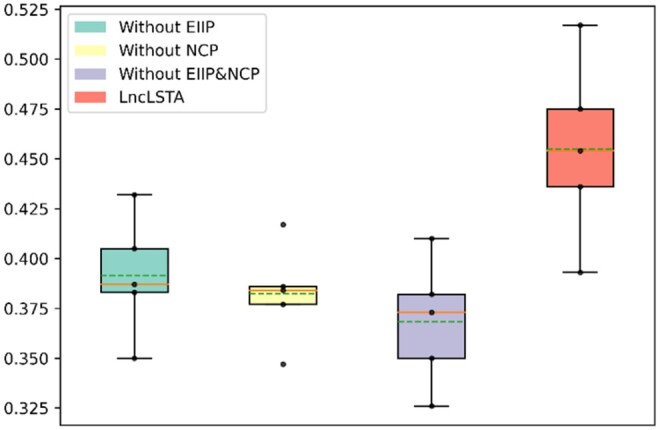
MaF1 comparison of feature ablation.

In this section, we do the ablation experiment to evaluate the effectiveness of our proposed input features and components of the model framework. We firstly conduct the ablation experiment on the feature extraction part for EIIP feature, NCP feature, and combination of EIIP and NCP features, respectively. Experimental results in Table 1, Fig. 2, and Supplementary Fig. S4 show that each feature input plays an important role in the overall prediction performance of the model. Notably, the most significant performance degradation of the model is observed when both EIIP and NCP features are eliminated. The model without any feature ablation shows the improvement on miAUC, maAUC, maF1, and ACC compared with the elimination of EIIP features and NCP features, by 0.008, 0.012, 0.084, and 0.024, respectively. These results indicate that each input feature of the model plays an important role in its overall effectiveness.

**Table 1.** Comparison of feature ablation experiments.^a^

	miAUC	maAUC	maF1	ACC	miAUPRC
Without EIIP	0.836 ± 0.012	0.735 ± 0.024	0.391 ± 0.041	0.555 ± 0.021	0.556 ± 0.016
Without NCP	0.837 ± 0.011	0.737 ± 0.021	0.382 ± 0.035	0.554 ± 0.026	0.559 ± 0.018
Without EIIP&NCP	0.833 ± 0.013	0.733 ± 0.023	0.368 ± 0.042	0.552 ± 0.024	0.554 ± 0.016
LncLSTA	**0.841 ± 0.009**	**0.745 ± 0.034**	**0.455 ± 0.062**	**0.576 ± 0.019**	**0.581 ± 0.019**

a Note that for maF1, it assigns equal weights to all classifications and doubles the multiplication of precision and recall in the computation. This would cause a notable fluctuation in imbalanced datasets.

The bold values represent the highest values for each evaluation metric.

Then we conduct the ablation experiment for the neural network components, as shown in Supplementary Table S1 and Supplementary Fig. S5. For the CM module, in order to ensure the feature dimension remains unchanged when this module is ablated and the feature is successfully passed to the subsequent modules, we replace the feature from CM module by the encoding from DeepLncLoc (Zeng *et al.* 2022). The experimental results show that the prediction of the model decreases substantially after removing the CM module. Subsequently, we conduct the ablation experiments on the LSTA and Bi-LSTM components separately, yielding the prediction results with varying degrees of degradation, although it is slightly higher than the results obtained from ablating the CM module. Then, we ablate the whole sequence information extraction module (LSTA and Bi-LSTM), and the prediction performance of each evaluation index decreases significantly. Compared with ablating the whole sequence information extraction module, the complete model of LncLSTA improves 0.035, 0.063, 0.233, and 0.055 in miAUC, maAUC, maF1, and ACC, respectively. These experimental results show that each module in the model plays an important role. In addition, we replace the focal loss by the cross entropy loss for comparison, demonstrating that the focal loss is beneficial for prediction, as also shown in Supplementary Fig. S5.

### 3.2 Comparison with machine learning methods

We first compare the performance of LncLSTA and traditional machine learning methods (SVM, RF, LR, and NN) on the lncRNA classification task. For these machine learning methods, they typically use *k*-mer as feature input. We choose the optimal *k*-mer size and parameters in machine learning models to obtain the classification in each method. Under the same sequence input conditions, we use the 5-fold cross-validation for the performance comparison, as shown in Table 2.

**Table 2.** Comparison of LncLSTA and machine learning methods.

Model	miAUC	maAUC	maF1	ACC
SVM	0.793 ± 0.009	0.701 ± 0.021	0.218 ± 0.009	0.496 ± 0.019
RF	0.796 ± 0.013	0.715 ± 0.013	0.215 ± 0.006	0.481 ± 0.009
LR	0.696 ± 0.029	0.601 ± 0.018	0.219 ± 0.021	0.415 ± 0.017
NN	0.708 ± 0.044	0.612 ± 0.012	0.229 ± 0.009	0.402 ± 0.054
LncLSTA	**0.841 ± 0.009**	**0.745 ± 0.034**	**0.455 ± 0.062**	**0.594 ± 0.039**

The bold values represent the highest values for each evaluation metric.

**Figure 3.**
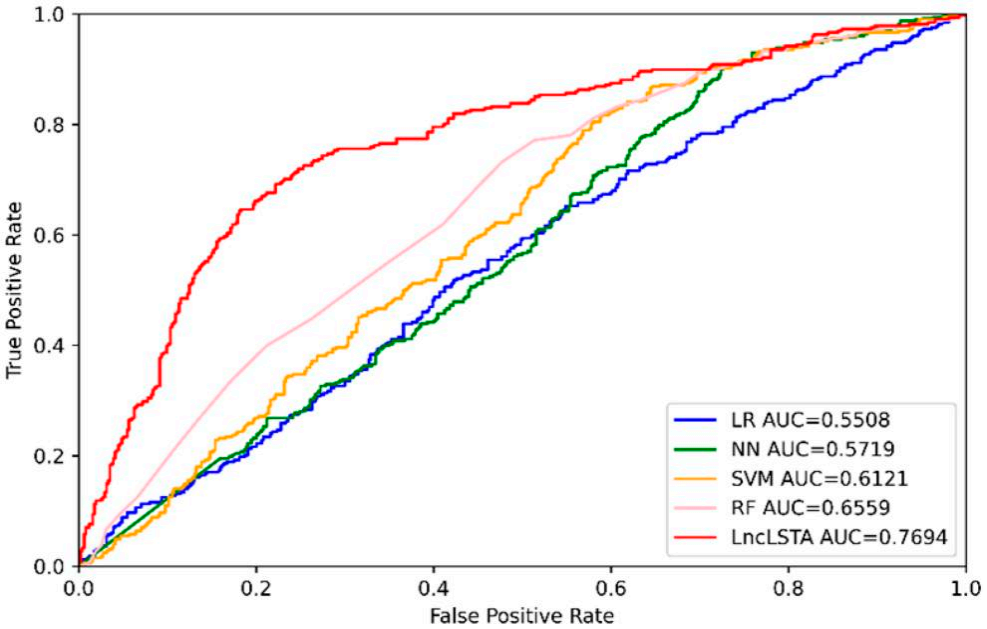
ROC curves comparing LncLSTA with other machine learning methods for cytoplasm subcellular localization.

We find that LncLSTA is significantly higher than other machine learning methods in all evaluation metrics. Figure 3 and Supplementary Fig. S6 plot the ROC curves of our method and other machine learning methods, from which it can be seen that LncLSTA outperforms other methods in all five subcellular localizations, especially in cytoplasm and ribosome. It slightly outperforms the NN method in exosome. Overall, these results demonstrate that LncLSTA outperforms traditional machine learning methods.

### 3.3 Comparison with other competitive lncRNA predictors

To further evaluate the performance of LncLSTA in predicting lncRNA subcellular localization, we compare it with other state-of-the-art predictors [lncLocator (Cao *et al.* 2018), iLoc-lncRNA (Su *et al.* 2018), iLoc-lncRNA2.0 (Zhang *et al.* 2022), DeepLncLoc (Zeng *et al.* 2022), and GraphLncLoc (Li *et al.* 2023)] using an independent test set. LncLocator and DeepLncLoc predict five subcellular localizations of lncRNAs, including Cytoplasm, Nucleus, Exosome, Ribosome, and Cytosol. iLoc-lncRNA, iLoc-lncRNA2.0, and GraphLncLoc predict four subcellular localizations of lncRNAs, including Cytoplasm, Nucleus, Exosome, and Ribosome.

The test set is combined from all experimental RNA subcellular localization dataset of lncSLdb and the screening of RNAlocate2.0. Because the lncSLdb database only collects five subcellular localizations (Nucleus, Chromosome, Cytoplasm, Nucleoplasm, and Ribosome), and it does not record other two localizations (Cytosol and Exosome), we randomly selected some samples from lncSLdb. The RNAlocate2.0 dataset contains a large number of multi-labeled lncRNAs. We filter out lncRNAs with only single labels, integrate two parts of these datasets and use the CD-HIT tool to remove redundant sequences with a cut-off of 80%. Finally, the independent test set consists of 91 Nucleus samples, 80 Cytoplasm samples, 46 Exosome samples, 17 Cytosol samples, and 10 Ribosome samples. Since iLoc-lncRNA and iloc-lncRNA2.0 treat Cytoplasm and Cytosol as one class, they predict only four classes with Cytoplasm, Nucleus, Exosome and Ribosome. So for the fair comparison, we categorize Cytosol into the Cytoplasm class when comparing LncLSTA with iloc-LncRNA and LncRNA2.0.

**Figure 4.**
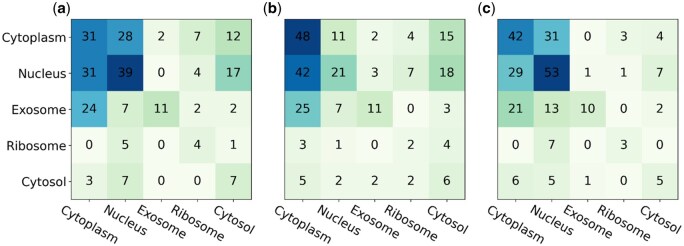
Confusion matrices of DeepLncLoc, lncLocator and LncLSTA for five classifications (a: DeepLncLoc, b: lncLocator, c: LncLSTA).

**Figure 5.**
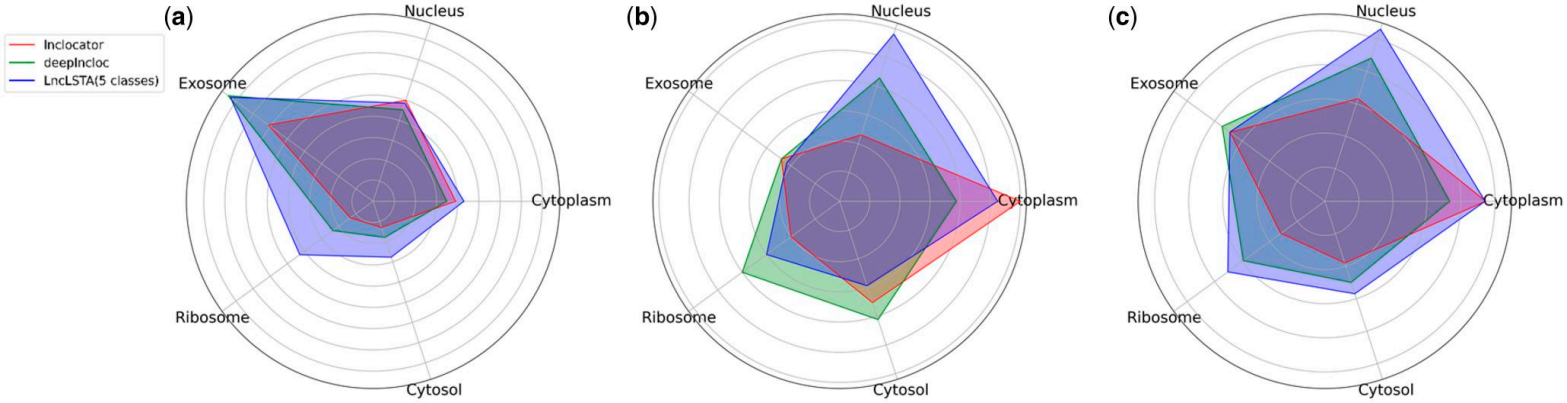
Precision, Recall, and F-measure of LncLSTA (five categories) with lnclocator, DeepLncLoc for each category on the test set (a: Precision, b: Recall, c: F-measure).

The detailed comparison between our LncLSTA and DeepLncLoc, lncLocator, iLoc-lncRNA, iLoc- LncRNA2.0, and LncLSTA is shown in Table 3. It demonstrates that LncLSTA outperforms these models in terms of accuracy, precision, recall, and F-measure. Specifically, in the 5-category prediction, LncLSTA correctly predicts a higher number of LncRNAs (113 out of 244) compared to DeepLncLoc (92) and lncLocator (82) (Fig. 4). Similarly, in the 4-category prediction, LncLSTA achieves a better performance, correctly predicting 122 LncRNAs out of 244, surpassing iLoc-lncRNA (118) and iLoc-lncRNA2.0 (102) (Supplementary Fig. S7). The analysis also reveals that LncLSTA performs well in handling imbalanced data, particularly for categories with smaller sample sizes like Ribosome and Cytosol. It consistently achieves high F-measure scores in subcellular localization of Nucleus, Ribosome, and Cytosol compared to other models (Supplementary Tables S2 and S3). Furthermore, a radar chart comparing several five-classification models shows that LncLSTA significantly outperforms others across all evaluation metrics (Fig. 5). Similarly, in the comparison of four-classification models, LncLSTA overwhelmingly outshines other two models in terms of accuracy, recall, and F-measure (Supplementary Fig. S8). In conclusion, the results demonstrate that LncLSTA is a powerful and superior model for predicting LncRNA subcellular localization compared to other state-of-the-art models. Its ability to handle imbalanced data and consistently achieve high performance makes it a valuable tool in this related field of research.

**Table 3.** Prediction performance comparison of LncLSTA with lncLocator, iLoc-lncRNA, iloc-lncRNA2.0, and DeepLncLoc on the test set.

Predictor	Macro Precision	Macro Recall	maF1	ACC
lncLocator(5 classes)	0.353	0.325	0.297	0.361
DeepLncLoc(5 classes)	0.412	0.373	0.345	0.377
iLoc-lncRNA(4 classes)	0.467	0.420	0.412	0.483
iLoc-lncRNA2.0(4 classes)	0.448	0.394	0.361	0.418
GraphLncLoc(4 classes)	0.410	0.303	0.324	0.429
LncLSTA(5 classes)	0.491	0.384	0.397	0.463
LncLSTA(4 classes)	0.577	0.446	0.463	0.500

### 3.4 Interpretability between lncRNA sequence motif and subcellular localization

**Figure 6.**
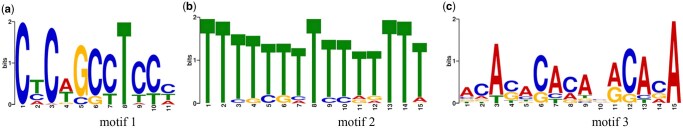
lncRNA motifs with subcellular localization correlation. (a) motif 1. (b) motif 2. (c) motif 3.

In this section, we use the meme method (https://meme-suite.org/) to conduct a comprehensive analysis of lncRNA sequence motifs and their correlation with subcellular localization in cells. When applying the meme method on the training set, we discover several sequence motifs exhibit correlations with corresponding subcellular localizations, as shown in Fig. 6. For example, the sequence motif 1 (“CTCAGCCTCCC”) in Fig. 6a displays an association with the subcellular localization of Ribosomes, accounting for 34.52% of the correlated sequences. In Fig. 6b, the motif 2 (“TTTTTTTTTTTTTTTT”) exhibits a correlation (29.46%) with the subcellular localization of Nucleus. This T-rich motif is known to be prevalent in eukaryotic genomes and has been implicated in functions related to the RNA stability and regulation, as suggested by its potential role in nuclear processes (Shrikumar *et al.* 2018). For the subcellular localization of Cytosol, the sequence motif 1 also has a high correlation of 17.8%. In addition, we find the sequence motifs most correlated with Exosome and Cytoplasm are motif 1 and motif 3 (“ACACACACACACACA”, shown in Fig. 6c), respectively, with a correlation of 7.4% and 7.9%, respectively. The ACACAC motif, a short tandem repeat commonly found in genomes, may contribute to the structural and functional organization of cellular components, potentially influencing the sorting and transport of RNAs and proteins within the cell (Ellegren 2004).

Furthermore, when utilizing LncLSTA to make predictions on the independent test set, we accurately predict 3 samples during the Ribosome localization prediction, with one of them containing the motif 1, showing a 33% correlation with Ribosome localization. In the Nucleus subcellular localization prediction, we correctly forecast 53 samples, out of which 14 samples contain the motif 2, corresponding to a 26.4% association with Nucleus localization. In the Cytosol subcellular localization prediction, we successfully predict 5 samples, with 1 sample including the motif 1, indicating a 20% relevance to Cytosol localization. In the Exosome subcellular localization prediction, we precisely predict 5 samples, among which 1 sample holds the motif 1, suggesting a 10% connection to Exosome localization. Lastly, in the Cytoplasm subcellular localization prediction, we accurately forecast 42 samples, with 1 sample containing the motif 3, demonstrating a 2.3% relevance to Cytoplasm localization.

In addition, we apply the Occlusion algorithm from Captum (https://captum.ai/api/occlusion.html) to calculate the attribution values of the discovered motifs, which can exhibit the correlation for the subcellular localization. As an example, the gene sequence of A230006K03Rik, which is localized in Cytoplasm, has been analyzed. The results, as depicted in Supplementary Fig. S9, indicate that a specific motif sequence “ACACACACACACACA” (motif 3) has a high attribution value within this gene sequence, suggesting a correlation with its Cytoplasmic localization. Furthermore, we have identified another subsequence “GGACAAGGAGGACCA” that significantly influences the localization of A230006K03Rik. These results offer the interpretability between the lncRNA sequence motifs and subcellular localizations, which is helpful for providing valuable insights of the role of lncRNAs in subcellular localization and functional regulation.

**Figure 7.**
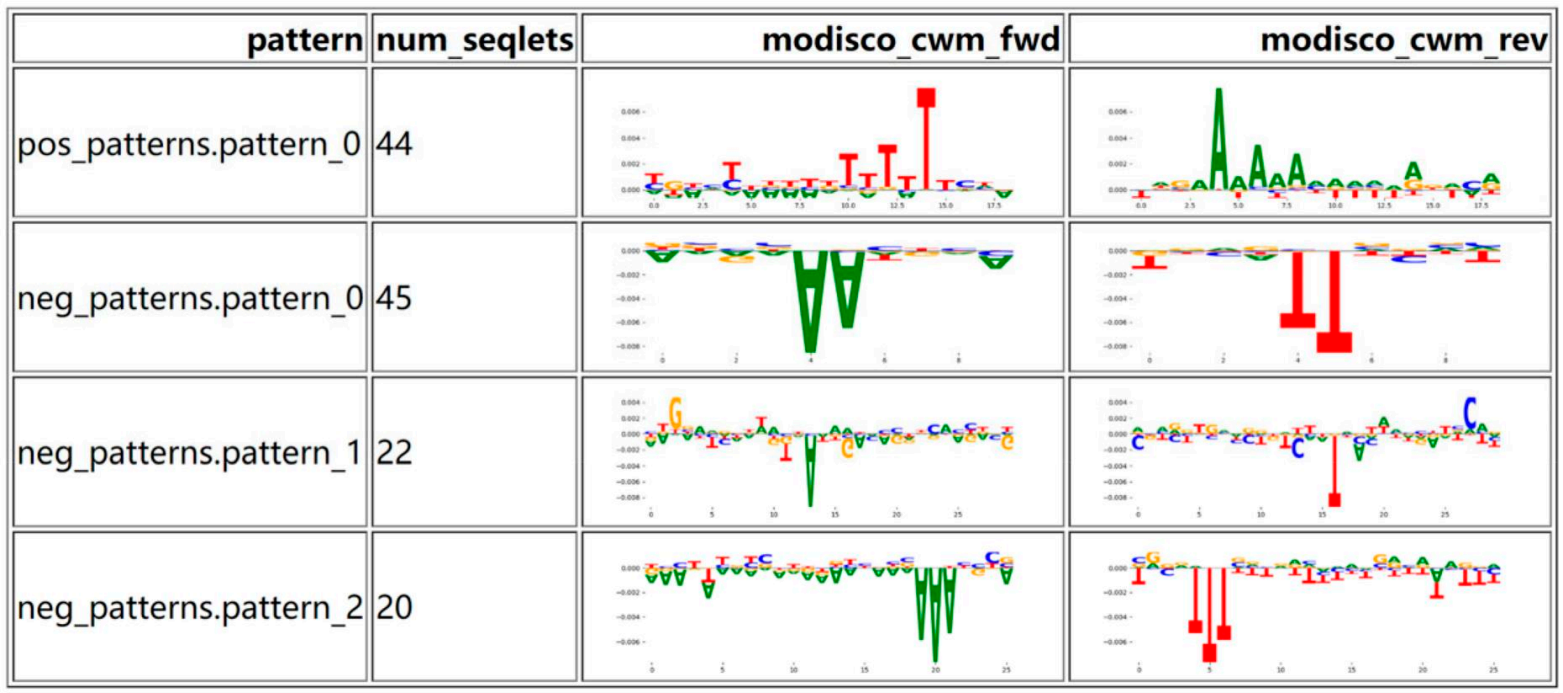
TFModisco-lite identified cytoplasmic lncRNA sequence motifs.

Finally, we utilize the SHAP library in Python with the KernelExplainer function to obtain feature importance scores for subcellular localization categories. These scores help us understand the impact of each feature on the predictions. Following this, we apply the TF-MoDISco-lite tool (Vejnar *et al.* 2019) to analyze sequence motifs associated with cytoplasmic localization. The analysis result is illustrated in Fig. 7 which reveals the key sequence patterns that play a crucial role in predicting cytoplasmic localization. For example, it shows at least one pattern (pos_patterns.pattern_0) being significantly enriched in cytoplasmically localized lncRNAs, suggesting that this specific sequence pattern may be associated with the function or stability of lncRNAs in the cytoplasm. The remaining motifs (neg_patterns.pattern_0, pattern_1, pattern_2) appear in the negative samples and do not show significant binding signals related to cytoplasmic localization.

### 3.5 Comparison with subcellular localization predictor of mRNAs

To explore the robustness, generalizability (transfer learning ability) of our LncLSTA, we select mRNA from other RNA species as a comparative benchmark for the subcellular localization prediction. As the most abundant class of RNAs, mRNA has been extensively studied in the field of subcellular localization, but we find current predictors for the subcellular localization hardly achieve the satisfying performance for both lncRNAs and mRNAs.

We choose the dataset from DM3Loc (Wang *et al.* 2021a,b) and do the comparison between our LncLSTA and DM3LOC, which achieves the best performance among current mRNA predictors. The dataset contains 17 870 mRNAs, among which 12 089 are localized in the nucleus, 17 704 in the exosomes, 2344 in the cytosol, 5293 in the ribosomes, 3256 in the membrane, and 1996 in the ER.

When applying our LncLSTA for the subcellular localization prediction of mRNAs, we only modify the model output by using a sigmoid function instead of a softmax function to output a probability value for each locus, which ranges from 0 to 1. We also optimize the loss function for the multi-label classification problem by improving the original focal loss function for multiple classifications. Specifically, we set an independent α value for each classification, where the size of α is proportional to the number of negative samples, i.e. the larger the α value, the greater the number of negative samples and the greater the weight to be given to the positive samples, so as to solve the problem of unbalanced distribution of positive and negative samples in some categories. This multi-label classification focal loss function can better adapt to the variability between different categories and improve the performance of the prediction model.

We evaluate the performance between LncLSTA and DM3Loc using three commonly used performance metrics: AUROC, AUPRC and MCC. According to Supplementary Table S4, we find LncLSTA achieves a relatively high AUROC and AUPRC, indicating that LncLSTA has a strong predictive power for the subcellular localization. Meanwhile, the MCC of LncLSTA in the nucleus reached 0.3664, indicating that the prediction results of the method are more reliable compared to DM3LOC.

The classification performance of LncLSTA is better compared with DM3LOC in classifications with a smaller number of samples, but slightly lower in classifications with a larger number of samples. We speculate that this may be due to the loss function adopted by LncLSTA, which performs well when dealing with a large number of negative samples, but does not perform well when dealing with a large number of samples. Compared with DM3Loc, LncLSTA has higher mean values for both AUROC and AUPRC. For example, there are 0.0037 and 0.0015 improvements on AUROC and AUPRC for each subcellular locus. LncLSTA, however, performs worse on the MCC metric relative to DM3Loc, which may be due to different weight assignments for misclassification by LncLSTA and DM3Loc in the loss function calculation. In summary, these experimental results demonstrate that the LncLSTA model has strong predictive ability and reliability in different subcellular localizations on not only lncRNAs but also mRNAs.

### 3.6 Discussion

While the LncLSTA model has demonstrated excellent performance, there remains room for improvement. We would like to discuss certain limitations of the LncLSTA model:

1. In this study, we trained and predicted on a specific subcellular localization of lncRNAs. However, it is essential to acknowledge that many lncRNAs can localize in multiple subcellular compartments. Therefore, we aspire to enhance the model's robustness in future research by collecting a more diverse set of lncRNAs with multiple subcellular localizations.
2. Our main focus in this article has been on the subcellular localization of lncRNAs, neglecting the subcellular localization of other RNA types. In future studies, we aim to integrate subcellular localization information for various RNA types, enabling more accurate predictions.

While existing computational methods struggle with the variable-length nature of lncRNA sequences, our research has yielded promising results by using the CM module. Although our study is confined to predicting the subcellular localization of lncRNAs, we believe that representing RNA and DNA sequences using the CM method could serve as a universal approach. This approach holds the potential to address variable-length sequence problems in other related domains, such as mRNA subcellular localization prediction, DNA N4-methylcytosine site prediction, RNA structure prediction, and transcription factor binding site prediction.

## 4 Conclusion

This article proposes an extensible deep learning model called LncLSTA, which aims to accurately predict the subcellular localization of lncRNAs. It firstly encodes the lncRNA sequences using various representations such as one-hot encoding, EIIP and NCP. By inputting these sequence encodings into the CM module, it can dynamically capture information around each nucleotide and extract feature. Next, it applies the LSTA module and Bi-LSTM network in the sequence information extraction module to effectively capture long-distance feature information. Finally, it inputs the extracted feature into the TextCNN module to achieve the final classification. Experimental results show that LncLSTA has great advantages in various indicators compared to other state-of-the-art models. In addition, LncLSTA can also be extended to predict the subcellular localization of mRNAs, achieving satisfactory prediction results. Based on our LncLSTA, we have developed an online web server (https://bis.zju.edu.cn/LncLSTA) to provide the subcellular localization prediction of LncRNAs and mRNAs.

## Supplementary Material

vbae173_Supplementary_Data
