# Supplementary material for: LncLSTA: a versatile predictor unveiling subcellular localization of lncRNAs through long-short term attention
Source: Bioinform Adv. 2024 Nov 22;5(1):vbae173. doi: 10.1093/bioadv/vbae173 (PMC11700581; doi:10.1093/bioadv/vbae173)
Supplement: vbae173_Supplementary_Data [file vbae173_supplementary_data.zip › Supplementary file-11.3.docx]

**LncLSTA: A versatile predictor unveiling the subcellular localization of lncRNAs through long-short term attention**

**(Supplementary materials)**

**Table S1**. Network component ablation experiments

|  | miAUC | maAUC | maF1 | ACC | miAUPRC |
| --- | --- | --- | --- | --- | --- |
| Without CM | 0.813±0.010 | 0.701±0.027 | 0.289±0.068 | 0.548±0.032 | 0.556±0.016 |
| Without LSA | 0.832±0.009 | 0.722±0.031 | 0.355±0.059 | 0.556±0.031 | 0.559±0.018 |
| Without BiLSTM | 0.829±0.011 | 0.738±0.032 | 0.341±0.054 | 0.558±0.020 | 0.561±0.016 |
| Without LSA&BiLSTM | 0.806±0.008 | 0.682±0.021 | 0.222±0.058 | 0.521±0.032 | 0.531±0.017 |
| Without Focalloss | 0.836±0.007 | 0.740±0.033 | 0.430±0.061 | 0.568±0.016 | 0.572±0.020 |
| LncLSTA | **0.841±0.009** | **0.745±0.034** | **0.455±0.062** | **0.576±0.019** | **0.581±0.019** |

**Table S2**. Precision, recall and F-measure of LncLSTA (5 categories) with Lnclocator, Deeplncloc for each category on the test set.

| Predictor | Subcellular location | Precision | Recall | F-measure |
| --- | --- | --- | --- | --- |
| Lnclocator | Cytoplasm | 0.390 | **0.6** | **0.473** |
|  | Nucleus | **0.5** | 0.231 | 0.316 |
|  | Exosome | 0.611 | **0.239** | 0.344 |
|  | Ribosome | 0.133 | 0.2 | 0.16 |
|  | Cytosol | 0.130 | 0.353 | 0.190 |
| Deeplncloc | Cytoplasm | 0.348 | 0.388 | 0.367 |
|  | Nucleus | 0.453 | 0.429 | 0.441 |
|  | Exosome | **0.846** | 0.239 | **0.373** |
|  | Ribosome | 0.235 | **0.4** | 0.296 |
|  | Cytosol | 0.179 | **0.412** | 0.25 |
| LncLSTA  (5 classes) | Cytoplasm | **0.429** | 0.525 | 0.471 |
|  | Nucleus | 0.486 | **0.582** | **0.53** |
|  | Exosome | 0.833 | 0.217 | 0.345 |
|  | Ribosome | **0.428** | 0.300 | **0.352** |
|  | Cytosol | **0.277** | 0.294 | **0.285** |

**Table S3**. Precision, recall and F-measure of LncLSTA quadruple classification with iLoc-lncRNA, iloc-lncRNA2.0 , GraphLncLoc for each category on the test set.

| Predictor | Subcellular location | Precision | Recall | F-measure |
| --- | --- | --- | --- | --- |
| iLoc-lncRNA | Cytoplasm | 0.473 | **0.639** | **0.543** |
|  | Nucleus | **0.550** | 0.418 | 0.475 |
|  | Exosome | 0.714 | **0.326** | **0.448** |
|  | Ribosome | 0.130 | 0.300 | 0.182 |
| iLoc-lncRNA2.0 | Cytoplasm | 0.437 | 0.608 | 0.508 |
|  | Nucleus | 0.490 | 0.286 | 0.361 |
|  | Exosome | 0.765 | 0.283 | 0.413 |
|  | Ribosome | 0.103 | 0.400 | 0.163 |
| GraphLncLoc | Cytoplasm | 0.386 | 0.338 | 0.360 |
|  | Nucleus | 0.402 | 0.725 | 0.518 |
|  | Exosome | 0.700 | 0.152 | 0.250 |
|  | Ribosome | 0 | 0 | 0 |
| LncLSTA  (4 classes) | Cytoplasm | **0.495** | 0.495 | 0.495 |
|  | Nucleus | 0.468 | **0.648** | **0.544** |
|  | Exosome | **0.846** | 0.239 | 0.373 |
|  | Ribosome | **0.500** | **0.400** | **0.445** |

**Table S4**. Comparison of LncLSTA and DM3Loc for mRNAs.

| Method | Compartment | AUROC | AUPRC | MCC |
| --- | --- | --- | --- | --- |
| LncLSTA | Nucleus | 0.7688 | **0.8863** | 0.3664 |
|  | Exosome | **0.7422** | **0.9989** | **0.0832** |
|  | Cytosol | **0.7434** | **0.3211** | 0.2623 |
|  | Ribosome | 0.7492 | 0.5335 | 0.3445 |
|  | Membrane | 0.7449 | 0.4456 | **0.3211** |
|  | ER | **0.7233** | **0.2615** | **0.2110** |
|  | Average | **0.7453** | **0.5744** | 0.2647 |
| DM3Loc | Nucleus | 0.7725 | 0.8765 | **0.3859** |
|  | Exosome | 0.7233 | 0.9965 | 0.0736 |
|  | Cytosol | 0.7406 | 0.3193 | **0.2872** |
|  | Ribosome | **0.7589** | 0.5478 | **0.3550** |
|  | Membrane | **0.7558** | 0.4472 | 0.3115 |
|  | ER | 0.6981 | 0.2502 | 0.2048 |
|  | Average | 0.7415 | 0.5729 | **0.2696** |


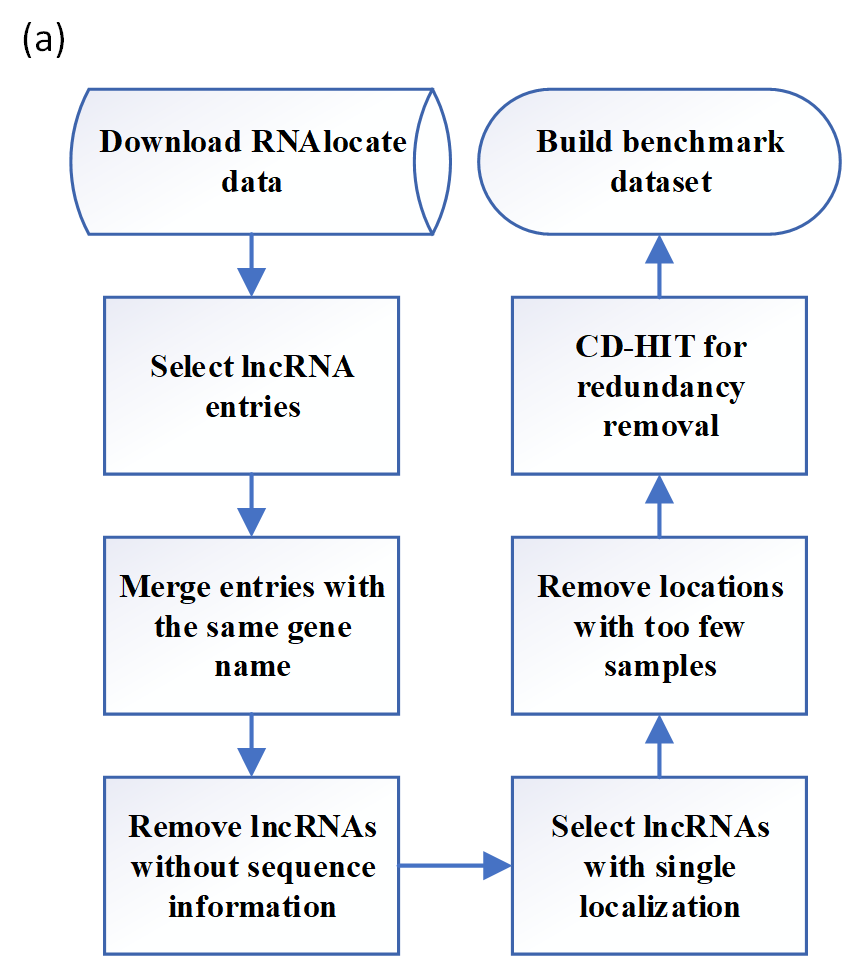

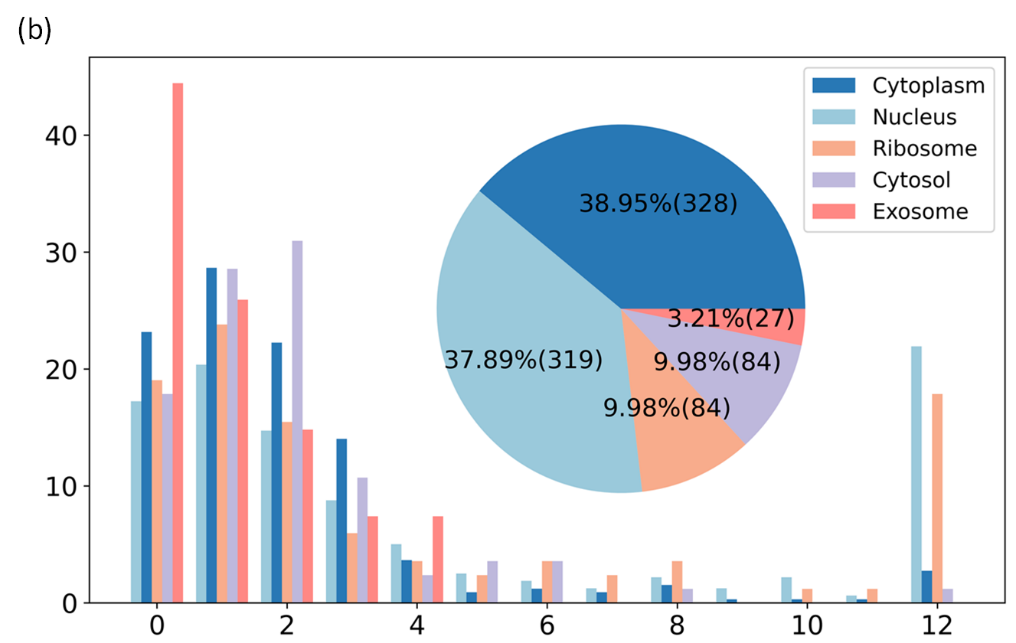


**Figure S1**. Data processing (a) and data distribution map of dataset (b).


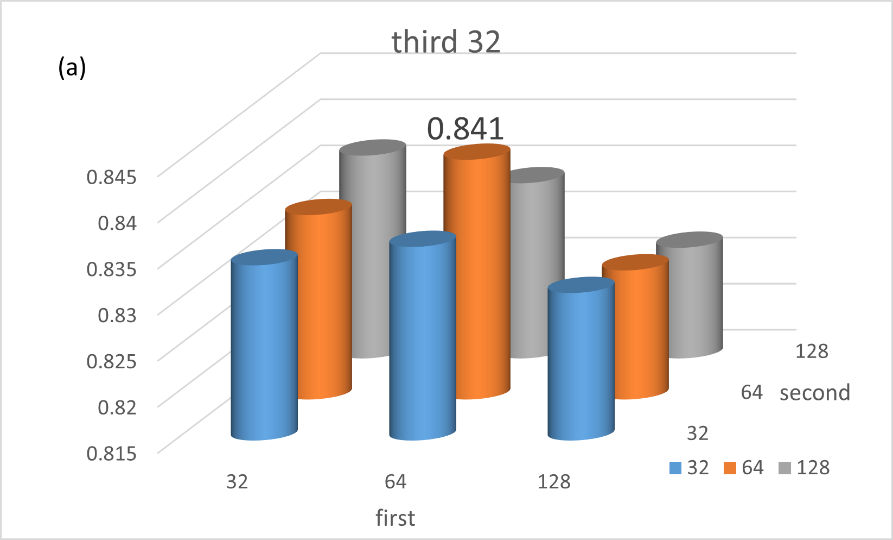

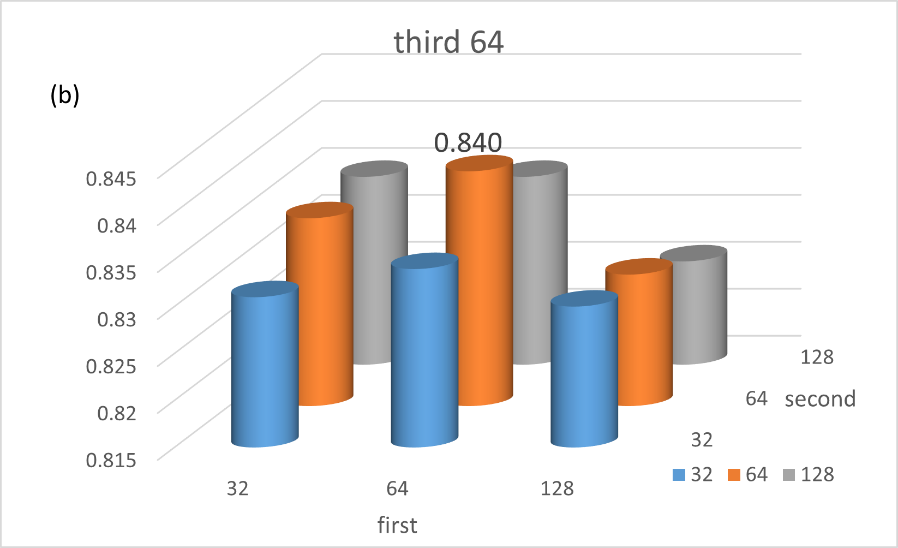


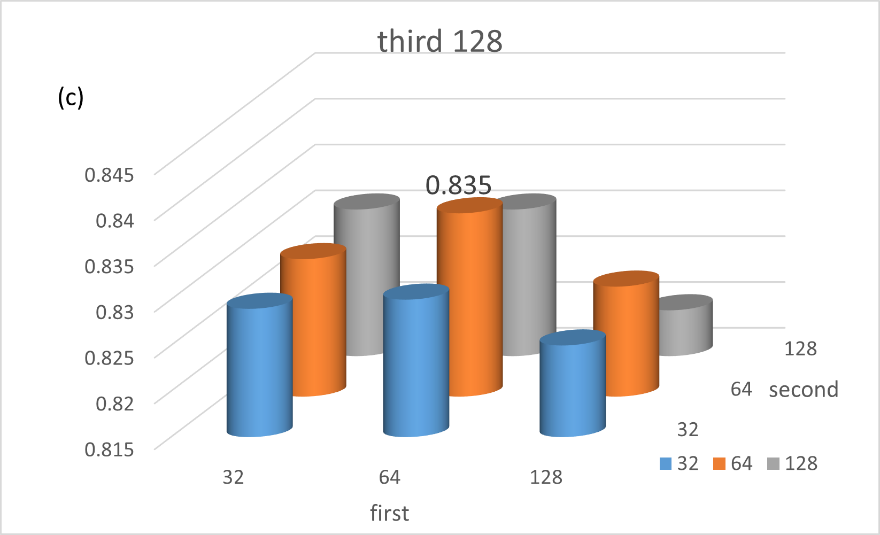


**Figure S2**. Results obtained by selecting different numbers of convolutional kernels (32, 64, and 128) in the CNN-maxpooling module. (Note that above three images demonstrate the results when performing three consecutive convolutional pooling operations with different numbers of convolutional kernels (32, 64, and 128). The first (a), second (b) and third (c) images showcase the outcomes by selecting 32, 64 and 128 convolutional kernels during the third convolutional pooling operation respectively, while choosing the number of kernels in the first and second convolutional operations involved with 32, 64 and 128. We find the model achieves the optimal prediction performance when selecting [64, 64, 32] as the number of three convolutional kernels.)


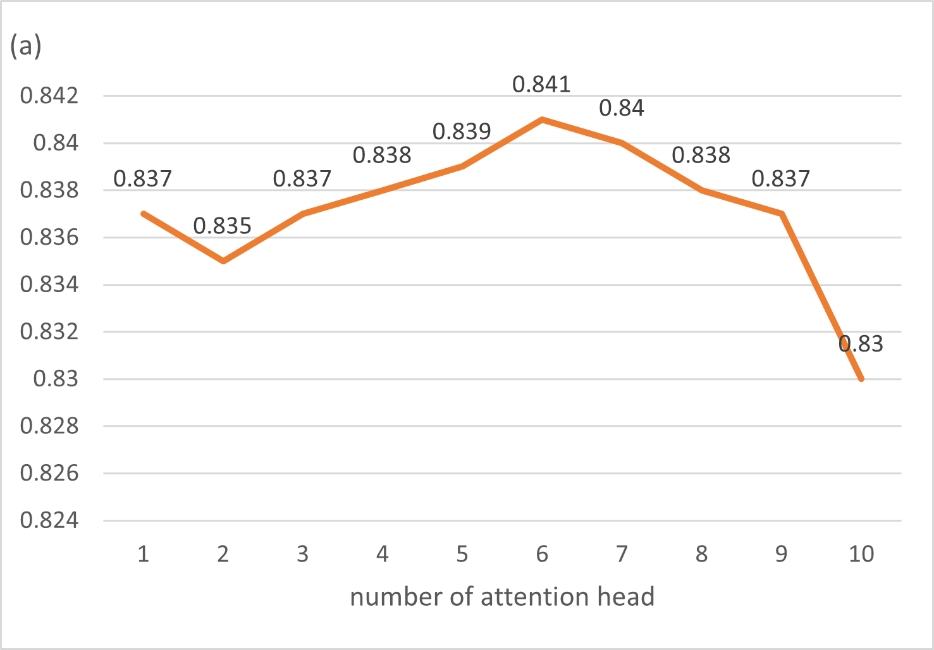

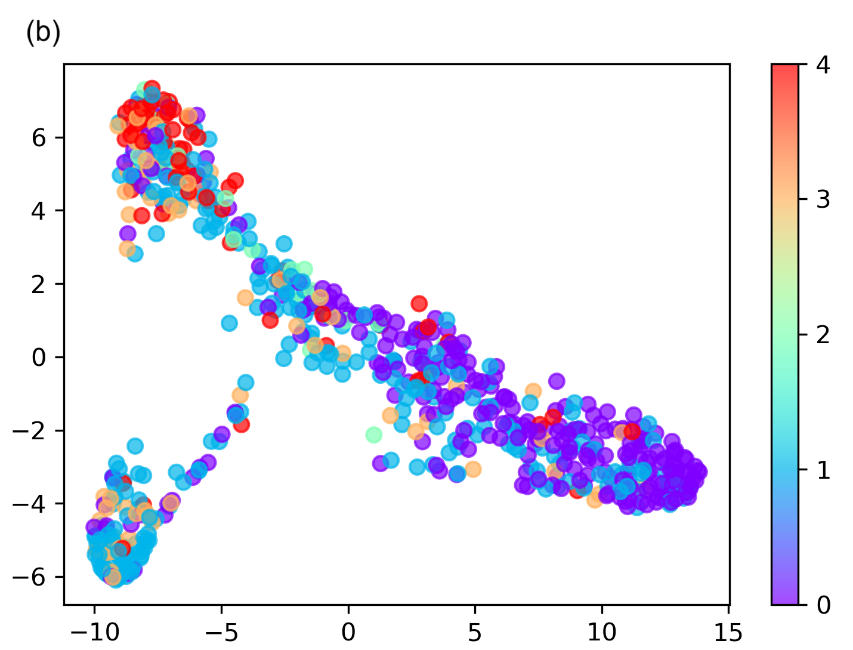


(a) Attention Heads Performance Analysis (b) T-SNE visualization when attention heads is set to 5


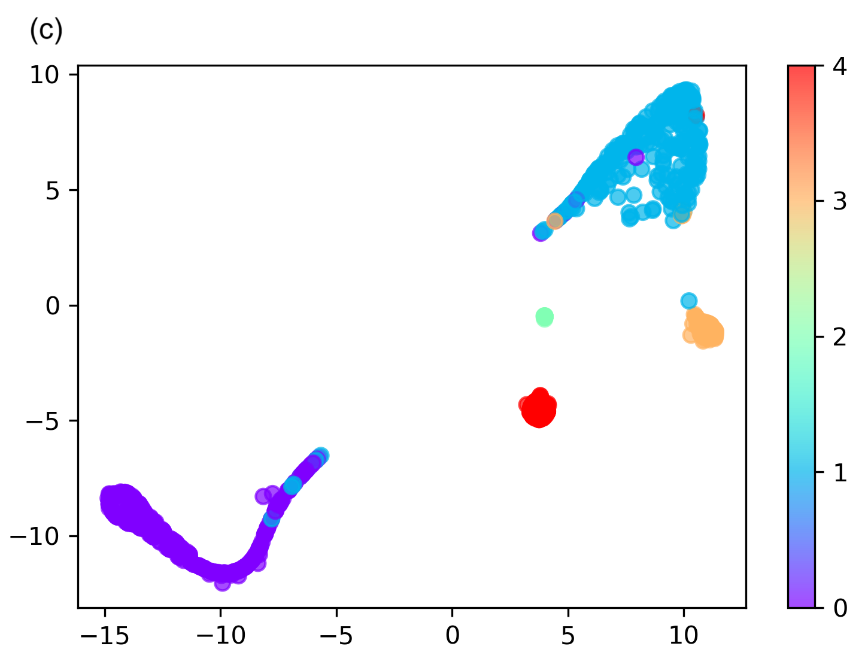

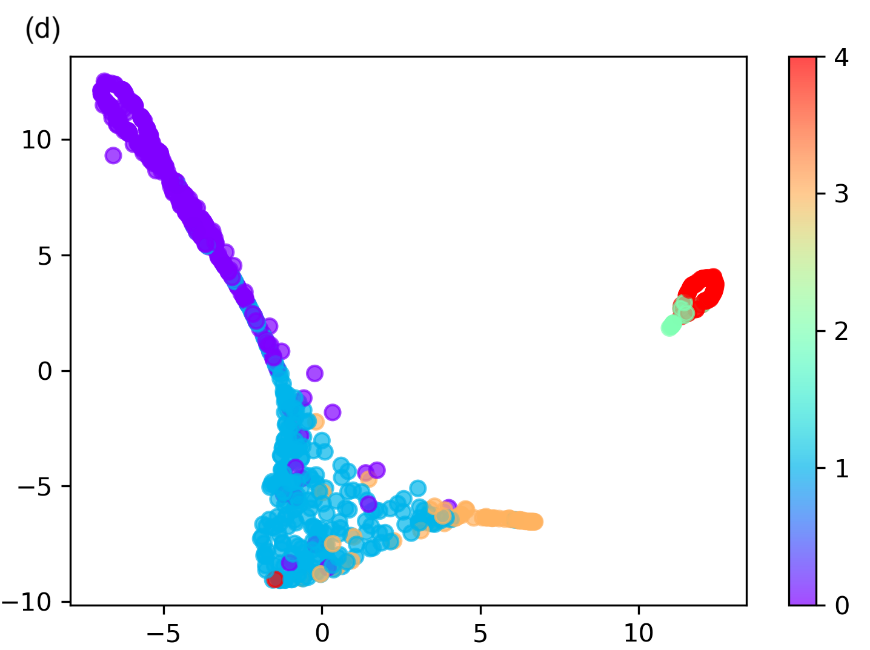


(c) T-SNE visualization when attention head is set to 6 (d) T-SNE visualization when attention heads is set to 7

**Figure S3**. Attention heads performance analysis and the T-SNE visualizations for different numbers of attention heads (5, 6, 7).

(Note that we conduct the result analysis on different numbers of attention heads in the LS term attention module and plot the miAUC curve with respect to the number of attention heads ranging from 1 to 10. From the results shown in (a), it can be observed that the optimal performance is achieved when the number of attention heads is 5, 6, and 7. Subsequently, we perform the T-SNE visualization on the output features using attention heads of 5, 6 and 7. According to the results depicted in (b), it can be seen that the T-SNE visualization of the output features is poor when the number of attention heads is 5. The best results were obtained with 6 attention heads, where the boundaries between different subcellular localizations are more distinct, as shown in (c). However, as the number of attention heads increases to 7, the performance showed a slight decline compared to the case with 6 attention heads, as shown in (d). Therefore, the number of attention heads is set to 6 in the LS term attention module.)


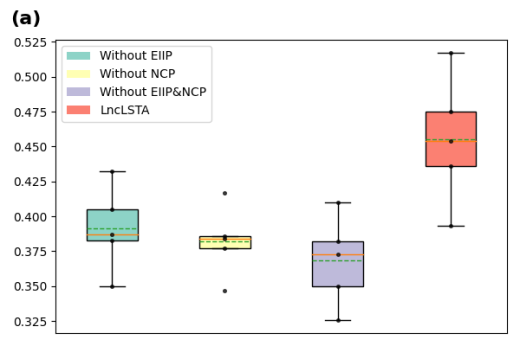

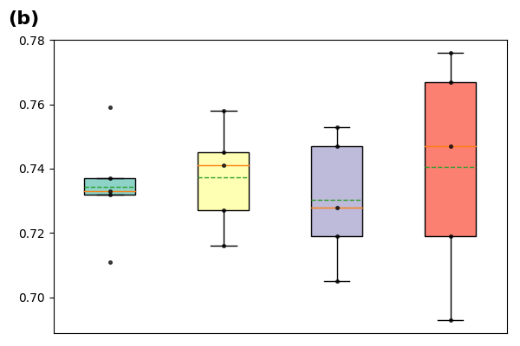


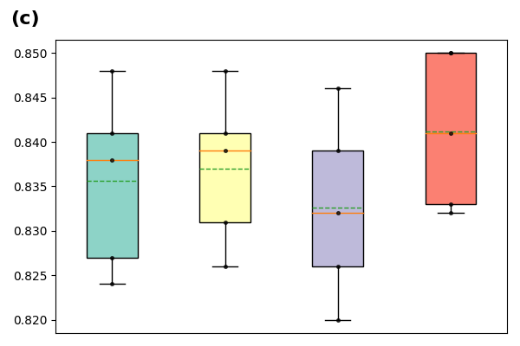

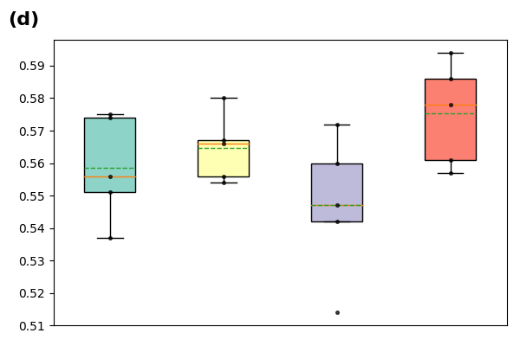


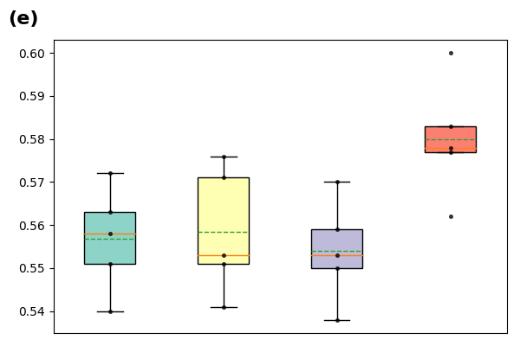


**Figure S4**. Comparison of input feature ablation experiments (a: maF1, b: maAUC, c: miAUC, d: acc, e: miAUPRC).


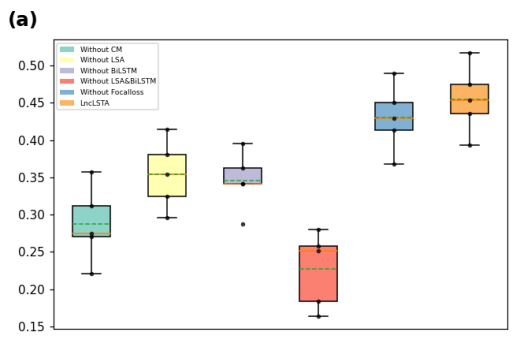

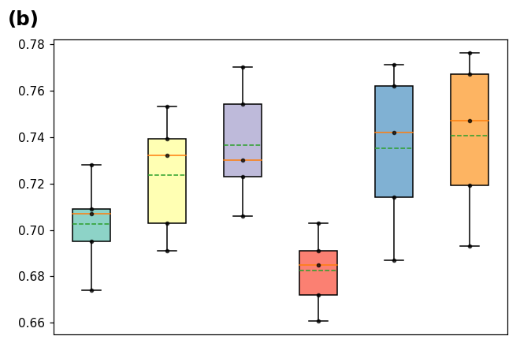


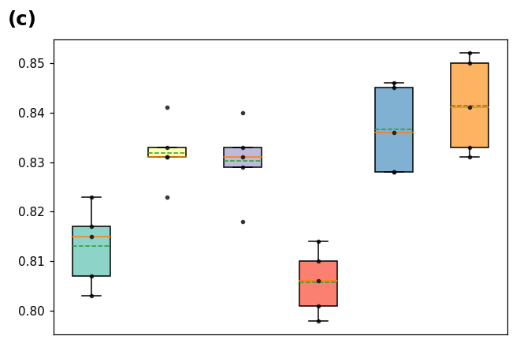

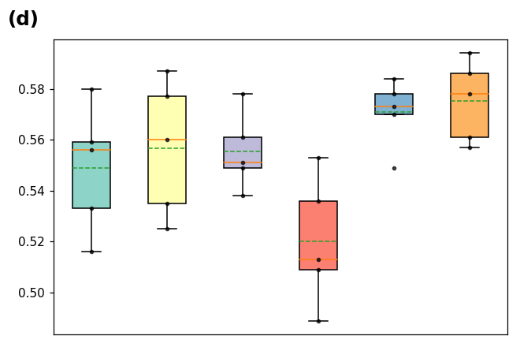


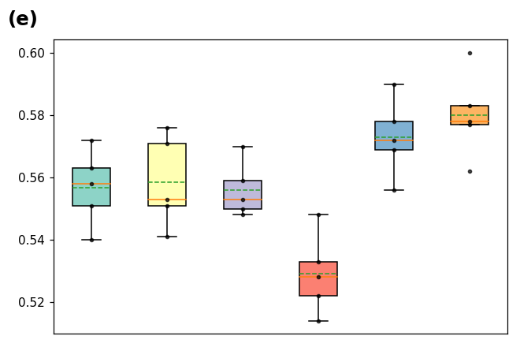


**Figure S5**. Component ablation experiments (a: maF1, b: maAUC, c: miAUC, d: acc, e: miAUPRC). Note that in these subfigures, without CM means the ablation of CNN-Maxpooling module, without LSA is the ablation of long-short term attention part, without BiLSTM is the ablation of BiLSTM part, without LSA&BiLSTM indicates that the whole sequence feature extraction module is ablated, without focal loss signifies the loss function is replaced by the cross entropy loss.


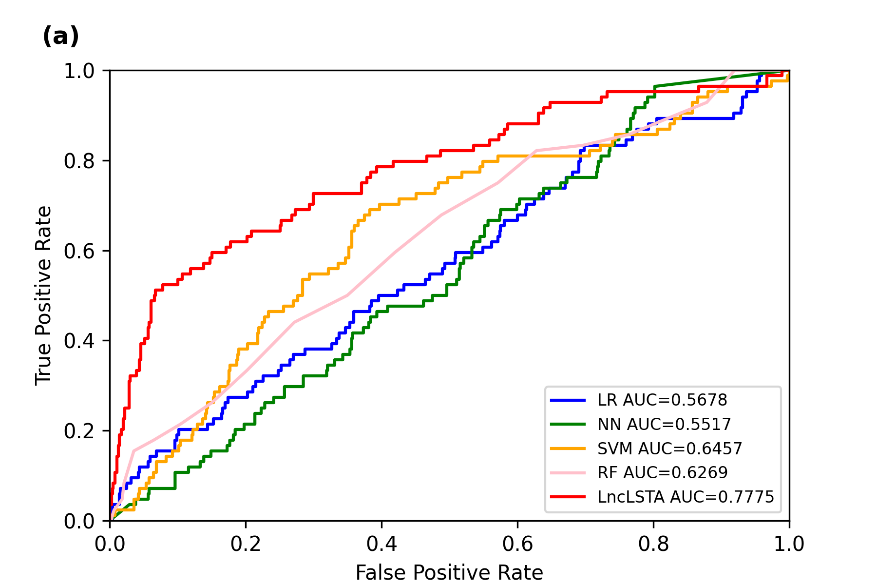

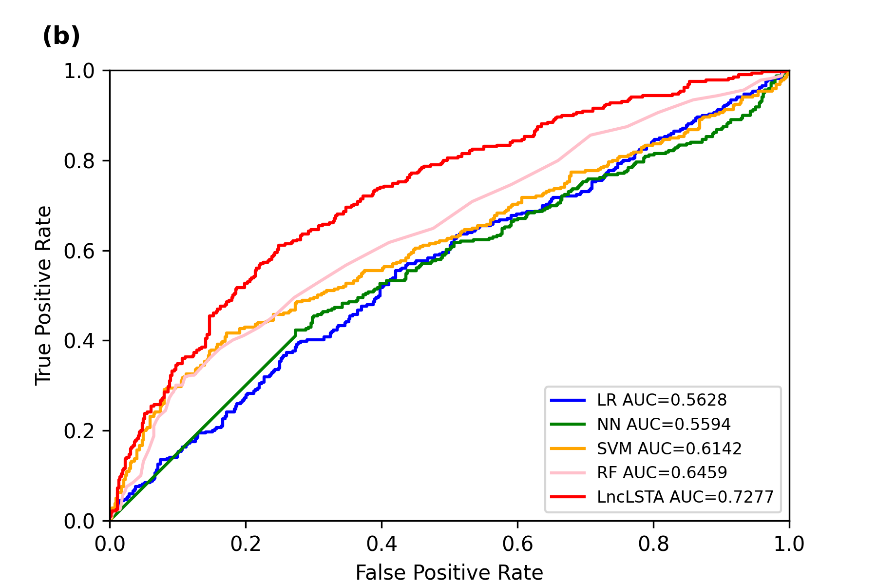


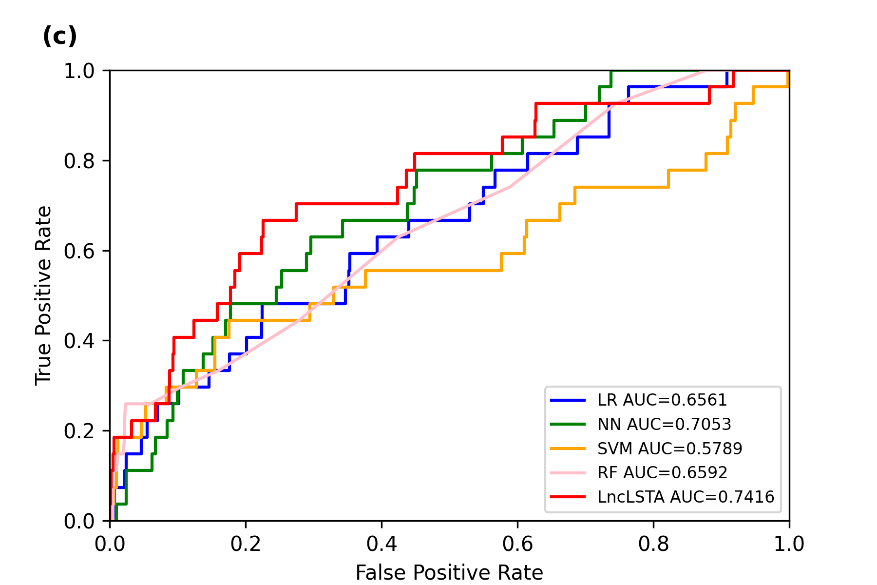

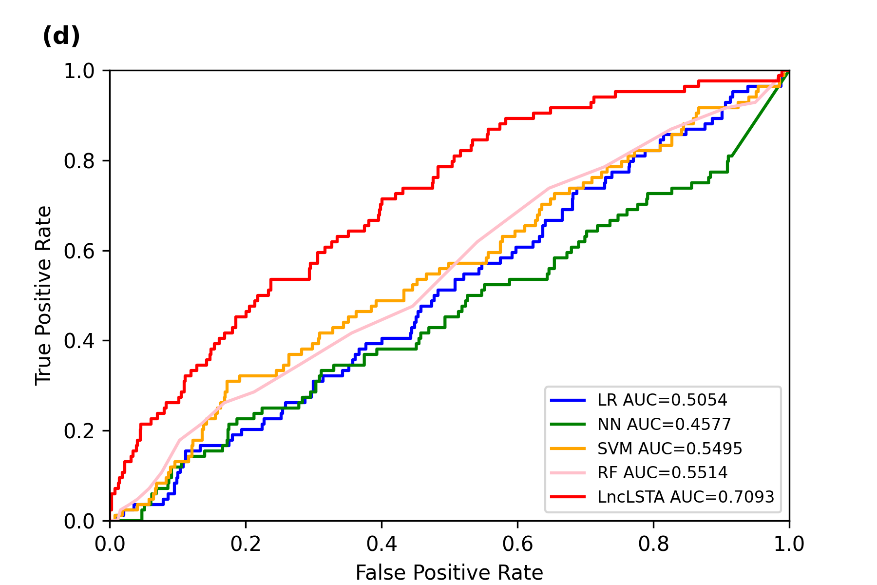


**Figure S6**. ROC curves comparing LncLSTA with other machine learning methods for other four subcellular localization predictions (a: Cytosol, b: Nucleus, c: Exosome, d: Ribosome).


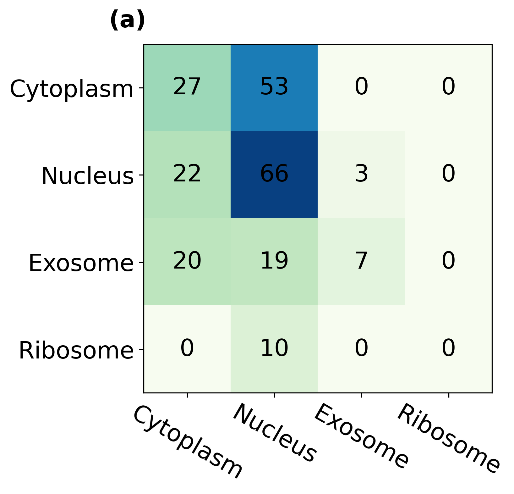

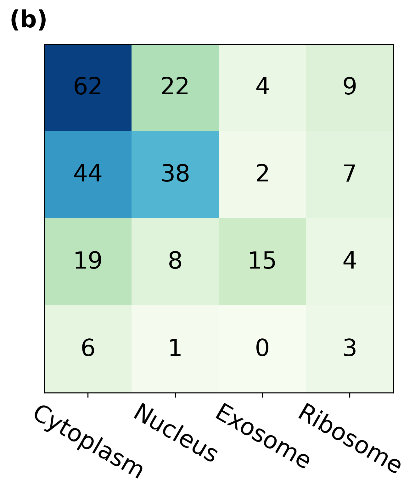

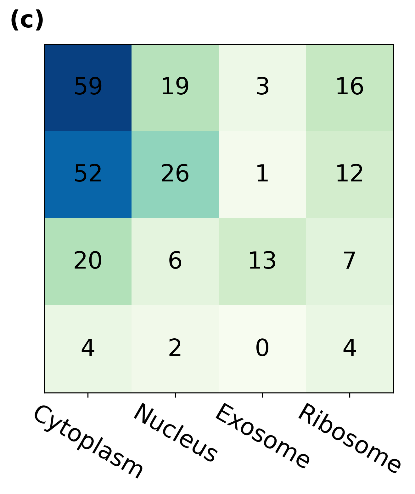

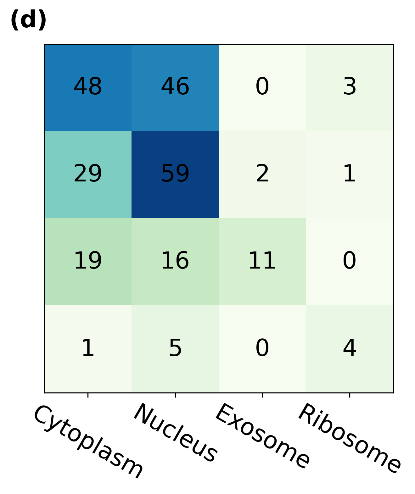


**Figure S7**. Confusion matrices of iloc_lncRNA, iloc_lncRNA2.0, GraphLncLoc and LncLSTA for four classifications (a: iloc_lncRNA, b: iloc_lncRNA2.0, c: GraphLncLoc, d: LncLSTA).


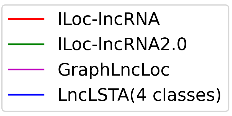

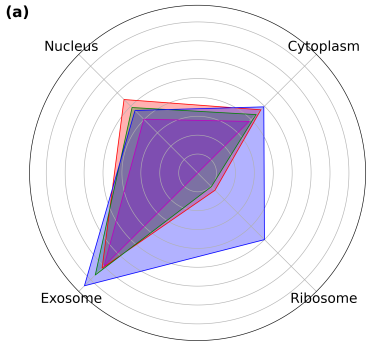

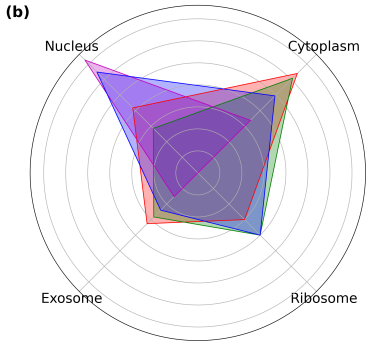

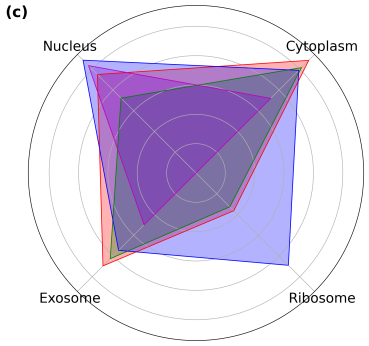


**Figure S8**. Precision, Recall and F-measure of LncLSTA (4 classifications) with iLoc-lncRNA, iloc-lncRNA2.0, GraphLncLoc for each category on the test set (a: Precision, b: Recall, c: F-measure).


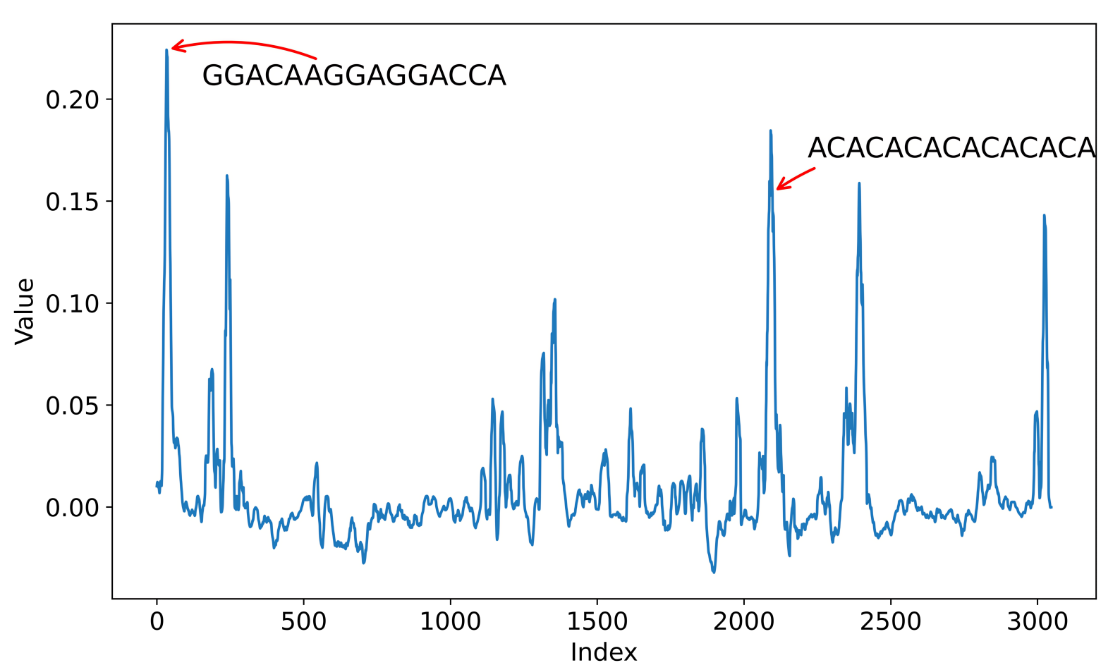


**Figure S9**. Result of attribution values for sequence of A230006K03Rik.
